# Impact of prenatal stress on offspring glucocorticoid levels: A phylogenetic meta-analysis across 14 vertebrate species

**DOI:** 10.1038/s41598-018-23169-w

**Published:** 2018-03-21

**Authors:** Zaneta M. Thayer, Meredith A. Wilson, Andrew W. Kim, Adrian V. Jaeggi

**Affiliations:** 1 Department of Anthropology, Dartmouth College, New Hampshire, USA; 2 Department of Anthropology, University of Illinois Urbana-Champaign, Illinois, USA; 3 Department of Anthropology, Northwestern University, Illinois, USA; 4 Department of Anthropology, Emory University, Georgia, USA

## Abstract

Prenatal exposure to maternal stress is commonly associated with variation in Hypothalamic Pituitary Adrenal (HPA)-axis functioning in offspring. However, the strength or consistency of this response has never been empirically evaluated across vertebrate species. Here we meta-analyzed 114 results from 39 studies across 14 vertebrate species using Bayesian phylogenetic mixed-effects models. We found a positive overall effect of prenatal stress on offspring glucocorticoids (d’ = 0.43) though the 95% Highest Posterior Density Interval overlapped with 0 (−0.16–0.95). Meta-regressions of potential moderators highlighted that phylogeny and life history variables predicted relatively little variation in effect size. Experimental studies (d’ = 0.64) produced stronger effects than observational ones (d’ = −0.01), while prenatal stress affected glucocorticoid recovery following offspring stress exposure more strongly (d’ = 0.75) than baseline levels (d’ = 0.48) or glucocorticoid peak response (d’ = 0.36). These findings are consistent with the argument that HPA-axis sensitivity to prenatal stress is evolutionarily ancient and occurs regardless of a species’ overall life history strategy. These effects may therefore be especially important for mediating intra-specific life-history variation. In addition, these findings suggest that animal models of prenatal HPA-axis programming may be appropriate for studying similar effects in humans.

## Introduction

Maternal effects are maternal influences on offspring phenotype that are independent of genotype^1^. Since the environment experienced during individual development may not be indicative of longer term trends, maternal effects allow organisms to developmentally adapt in response to a mother’s life-long signal of environmental experience^2,3^. While these effects have been reported in a wide range of species, the question of whether the capacity for maternal effects quantitatively varies across species varying in phylogeny and life history characteristics is not well understood. Such findings are important since they inform our understanding of how evolutionarily ancient this response may be, as well as under what ecological conditions such effects may be adaptive^4^. In addition, given the proposed role of maternal effects in the developmental origins of adult health and disease (DOHaD) hypothesis^5^, an important question is whether the use of animal models is helpful for understanding these effects in humans.

One of the most consistently studied exposures in the maternal effects literature is maternal stress. Maternal stress is an important signal of environmental conditions, and, when experienced prenatally, has been associated with morphological, behavioral, and physiological changes in a broad range of vertebrate offspring^6^. In a particularly impressive observational study that tracked the 10-year predator-prey cycle among snowshoe hares and lynxes, researchers found increased lynx density correlated with a reduction in birth size and more predator avoidant behaviors in hare offspring^7^. While maternal stress effects are sometimes interpreted as reflecting pathological impacts of prenatal stress exposure, such responses may also represent adaptive, fitness-enhancing adjustments for offspring and/or their mothers^4^. For example, being born into a high-predator environment could indicate elevated extrinsic mortality risk. Therefore maternal and offspring fitness may be enhanced by offspring adopting a faster life history strategy and investing in earlier reproduction, even if this comes at a cost to adult size, somatic maintenance, and life-span^8^.

The Hypothalamic Pituitary Adrenal (HPA)-axis is a physiological system that is hypothesized to be critical in mediating the relationship between prenatal stress and offspring developmental outcomes^9,10^. This evolutionarily-conserved system underlies the stress response across vertebrate species and is important for maintaining homeostasis^11–13^. Glucocorticoids, the end product of HPA-axis activation, are produced by the adrenal glands (or, in amphibians, by the interrenal glands) in response to adrenocorticotropic hormone secretion in the pituitary. Predator- and ecologically-induced stressors have been found to modify glucocorticoid levels, which in turn directly influence important life history variables such as maturation rate and body size^4,14–16^.

**Figure 1:**
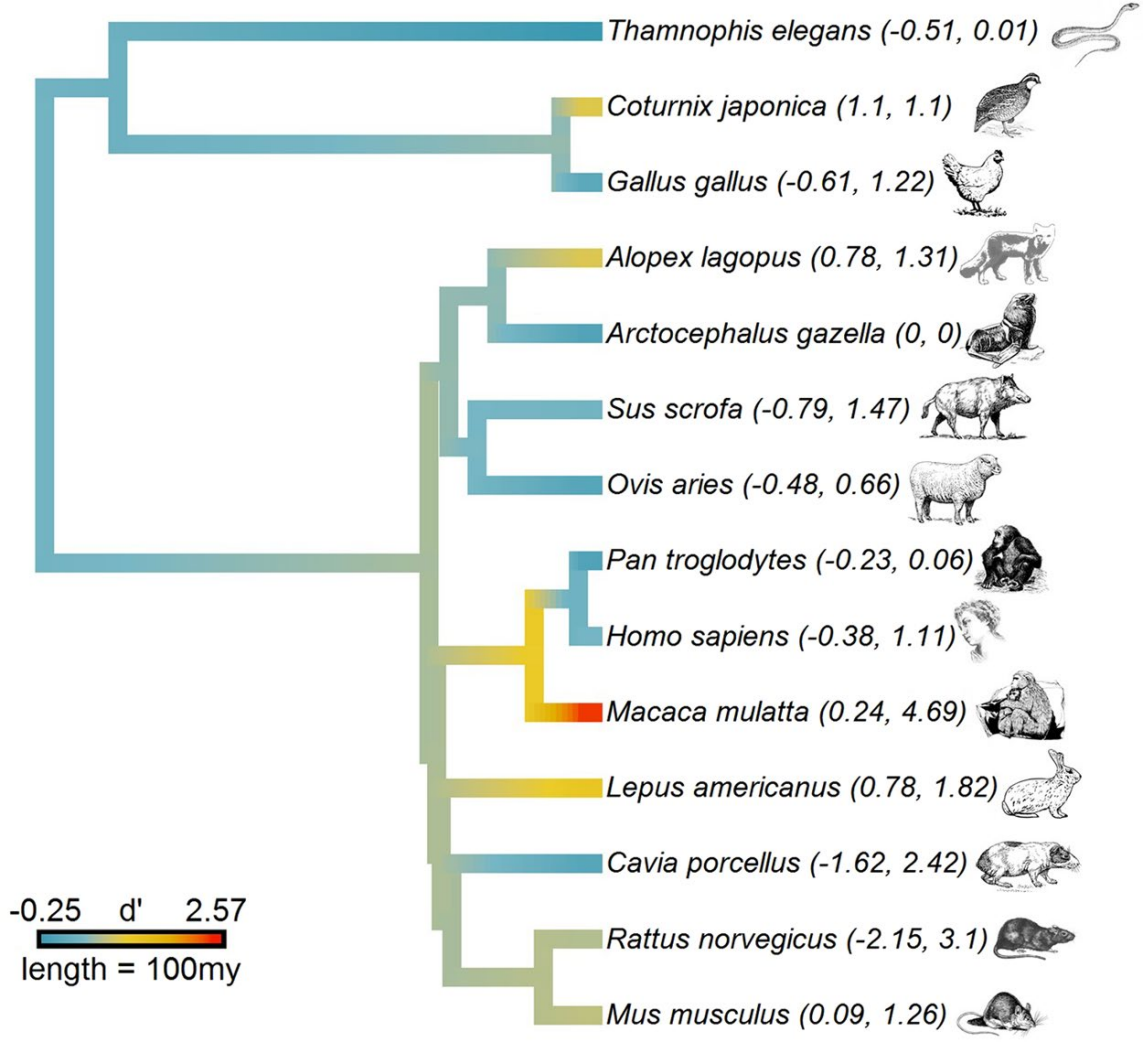
Phylogeny of study species with mean effect sizes for each species indicated by color and ranges given in parantheses. Internal branches are color-coded to reflect ancestral states inferred by maximum likelihood^54^. 100my = 100 million years.

While it is generally accepted that prenatal stress affects offspring glucocorticoids in humans and other vertebrates^17–20^, *to date no formal meta-analysis has been conducted to evaluate the empirical strength of this association across species*. Such an analysis will clarify whether programming occurs in all species, which has important implications for our understanding of the evolutionary origins of this response. Given this background, we here present a phylogenetic multilevel meta-analysis evaluating the relationship between prenatal stress and offspring glucocorticoid levels across a range of reptilian, avian, and mammalian species (*k* = 114 effect sizes from 39 studies on 14 species, see Fig. 1). Specifically, we were interested in determining (i) the overall effect size of prenatal stress on offspring glucocorticoids (i.e. whether there are differences in glucocorticoid baseline or glucocorticoid response to stress among individuals prenatally exposed to stress and/or exogenous glucocorticoids versus controls); (ii) whether there was a difference in offspring glucocorticoids based on moderators (see Table 1) such as offspring sex, timing, chronicity or severity of the stressor, experimental vs observational study design, and in the case of glucocorticoid reactivity - whether offspring stress exposure was mild or severe; and (iii) whether effect sizes differed across species, either as a function of phylogeny, being a mammal (and therefore prolonged exposure to maternal physiology), or life history characteristics. The results of this analysis have important implications for understanding the proposed universality of prenatal stress effects on glucocorticoid levels across vertebrates, as well as the environmental or study conditions that are most likely to induce such effects.

**Table 1:** Description of study variables in meta-regression.

Variable	Description
Baseline glucocorticoids	Measurement taken at specific time of day; if multiple times of day included, each coded as separate observation
Glucocorticoid reactivity	Peak glucocorticoid response to stress; the exact amount of time elapsed post stressor varied across studies
Glucocorticoid recovery	If only one measurement provided post-peak that measurement was used. If multiple measurements were provided we selected the time point with the greatest difference, if any, between case and control
Study design	Experimental vs observational
Offspring sex	Both when sexes were unspecified; when results listed separately by sex, classified as male or female
Timing of stress exposure	Early incubation/gestation (as defined by study authors) vs all other periods (mid/late gestation or across pregnancy)
Age of assessment	Neonate, juvenile, adult
Severity of maternal stressor	Defined as mild or extreme depending on severity of stressor. Used definition provided by study author when available; otherwise classified natural disasters, predator threat, synthetic glucocorticoid administration, and physical stressors as extreme and psychological stressors as mild
Severity of offspring stressor^a^	Defined as mild or extreme depending on severity of stressor. Used definition provided by study author when available; otherwise classified natural disasters, predator threat, and physical stressors as extreme and psychological stressors as mild
Mammal	Is the species a mammal or not? Included due to gestation providing more opportunities for programming in mammals, and Fig. 1 revealing an increase in effect size from the root of the tree to the ancestral mammal

^a^In cases of glucocorticoid reactivity/or recovery.

## Results

**Figure 2:**
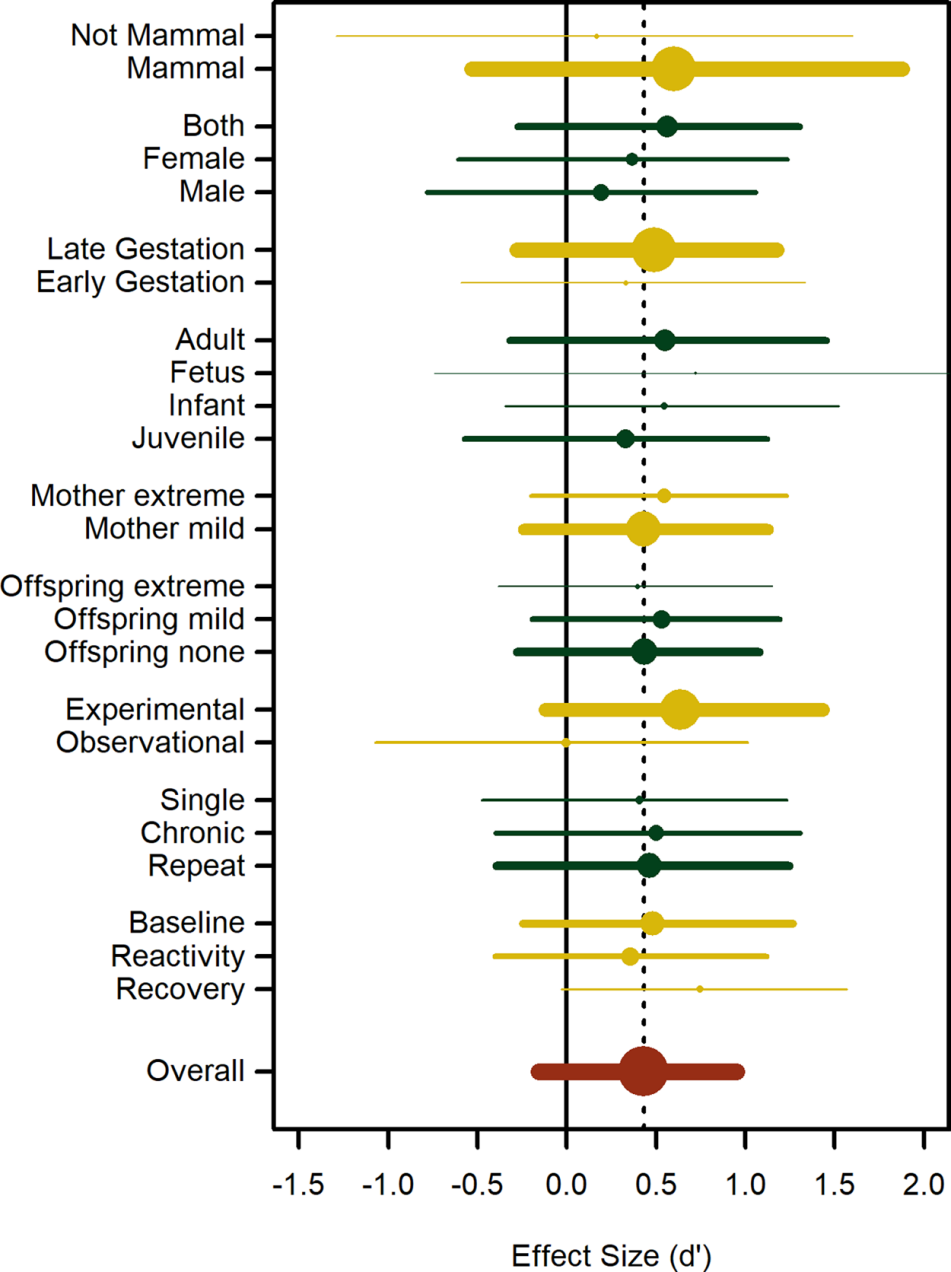
Overall weighted effect size and 95% Highest Posterior Density Intervals and the independent influence of each moderator variable in explaining variation in effect size. Experimental studies and those measuring glucocorticoid recovery stand out as producing higher effect sizes. Width of lines and size of points are proportional to the number of effect sizes in each category.

The overall weighted effect size of prenatal stress on later-life glucocorticoid physiology was positive, though the 95% credible intervals included 0 (*d’* = 0.431, 95% Highest Posterior Density Interval [HPDI] = −0.16–0.95, Posterior Probability [PP] _> 0_ = 0.94, Number of effect sizes [*k*] = 114, Fig. 2, see Supplementary Information for complete model results). Adding life history variables revealed that an increase in body size reduced the effect size (b = −0.72, 95% HPDI = −1.92–0.39, PP _< 0_ = 0.91, *k* = 114) while an increase in brain size increased it (b = 0.69, 95% HPDI = −0.54–1.92, PP _> 0_ = 0.89, *k* = 114), albeit both 95% HPDI’s overlapped with 0. Controlling for body and brain size we then examined the influence of each moderator in Table 1 independently. Figure 2 presents an overview of the results of these independent models. Most moderators did not have a strong influence, as different levels of the variables cluster tightly around the overall effect size (dashed line) and have wide, overlapping confidence intervals. However, effect sizes from experimental studies were more clearly different from 0 (*d’* = 0.64, 95% HPDI = −0.12–1.43, PP _> 0_ = 0.96, *k* = 93) than those from observational studies (*d’* = −0.01, 95% HPDI = −1.07–1.01, PP _> 0_ = 0.50, *k* = 21), and tended to be larger than those from observational studies (difference in *d’* = 0.64, 95% HPDI = −0.48–1.68, PP _> 0_ = 0.89). Similarly, effect sizes measured during recovery were more clearly different from 0 (*d’* = 0.75, 95% HPDI = −0.03–1.57, PP _> 0_ = 0.96, *k* = 17) than those measured during baseline (*d’* = 0.48, 95% HPDI = −0.24–1.26, PP _> 0_ = 0.93, *k* = 57) or peak reactivity (*d’* = 0.36, 95% HPDI = −0.40–1.12, PP _> 0_ = 0.88, *k* = 40), and the differences between recovery and baseline (difference in *d’* = 0.26, 95% HPDI = −0.14–0.64, PP _> 0_ = 0.91) and recovery and peak reactivity (difference in *d’* = 0.39, 95% HPDI = −0.01–0.77, PP_ > 0_ = 0.97) are quite likely to be positive. Thus, a model that includes both of these moderators fits the data substantially better (Deviance Information Criterion = −48.8) than the model with brain and body size only (DIC = −42.3); in this model, the estimated effect size of an experimental study measuring recovery is very likely to be greater than 0 (*d’* = 0.90, 95% HPDI = 0.09–1.78, PP _> 0_ = 0.98, *k* = 17). Pushing this point further, we constructed a full model that included all moderator variables and chose as the reference levels those that revealed the highest effect size in the independent models. Thus, the most propitious studies - which would expose mammal mothers to chronic extreme stressors in late gestation and assess glucocorticoid recovery after a mild stressor to fetal offspring of both sexes - would likely find very large effect sizes (*d’* = 2.216, 95% HPDI = 0.11–4.42, PP _> 0_ = 0.97); of course, no study in our sample had this exact configuration. However, in principle such a study could be designed.

**Figure 3:**
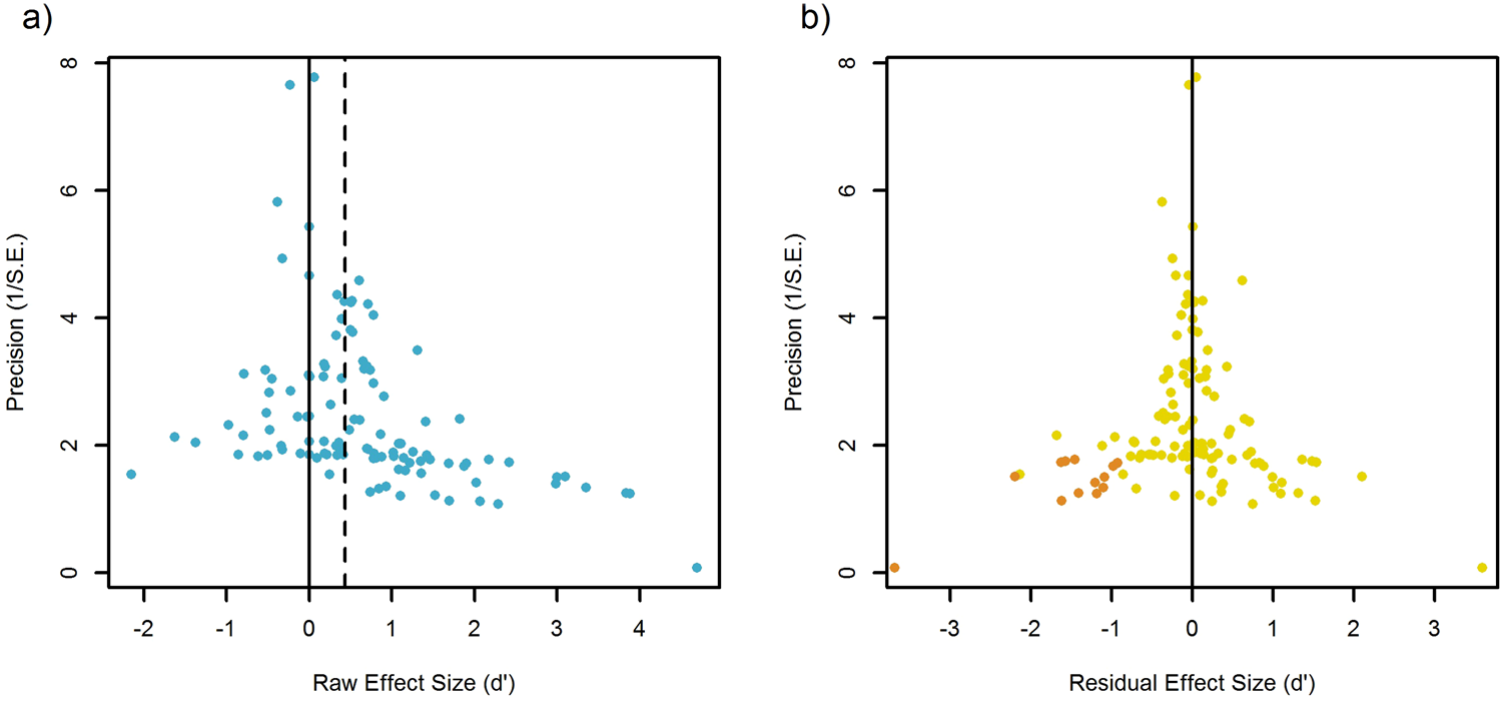
Funnel plots on (**a**) the raw effect sizes and (**b**) the residuals from the full model. Solid vertical line indicates zero and dashed vertical line indicates overall weighted effect size from the intercept-only model. Asymmetry in the funnel plot, specifically missing null or negative effects at low precision (lower left corner of the funnel), indicate publication bias. Egger’s regression on the residuals (**b**) revealed a trend towards publication bias, and the trim and fill method imputed 14 missing studies (brown points) in the lower left corner.

After accounting for variation in effect sizes due to all the moderators, our assessment of sources of heterogeneity in the full model found that the highest proportion of variance in effect sizes was explained by the study level random effect (0.16), followed by phylogenetic history (0.14) and species-level random effect (0.05). The intercept in Egger’s regression^21^ was likely different than 0 (PP_ > 0_ = 0.96), indicating unmeasured heterogeneity or publication bias; small studies with null or negative effect sizes are underrepresented, resulting in asymmetry in the funnel plot (see Fig. 3). Thus, following^22^ we used the trim-and-fill method^23^ (implemented in the R package *meta*^24^) on the residuals of the full model and their standard errors to estimate a corrected overall effect size (see Fig. 3b, see Methods for more detail). The trim and fill method added 14 missing studies and estimated the true effect size to be −0.058 (instead of 0); thus, all effect sizes should arguably be devalued by this amount^22^. This does not change our conclusions qualitatively.

Lastly, we explored the possibility of life history traits influencing effects of maternal stress among mammals only. Body size, brain size, gestation length, fertility, weaning age, age at first reproduction, and longevity were not significant moderators of effect size in mammals, whether they were all included in a full model, whether we used variance inflation factors to remove moderators with high collinearity (resulting in a model with fertility and body size only), or whether we selected specific traits that we hypothesized to be particularly important for maternal programming (gestation length, longevity; see SI).

## Discussion

This meta-analysis evaluating the relationship between prenatal stress and offspring glucocorticoids across vertebrates shows (i) a positive overall effect size, though with 95% credible intervals overlapping with 0, (ii) stronger effects for experimental studies (as opposed to observational ones) and those reporting glucocorticoid recovery (as opposed to baseline or peak reactivity), and (iii) little variation in effect sizes across species or life history characteristics. These findings indicate that offspring sensitivity to prenatal stress is found across vertebrates despite large differences in body size, maturation rate, and other aspects of life history, but that the strength of this association varies based on study design. It also suggests that animal models may be appropriate for studying DOHaD effects related to prenatal stress and their impacts on offspring stress physiology.

Even though prenatal stress was overall positively associated with later-life glucocorticoid physiology, the 95% credible intervals included 0. Given the large number of species, study designs, and the range of effect sizes (Fig. 1) - which introduce heterogeneity and thereby increase the confidence intervals of the overall effect size - this finding is somewhat expected. More research with comparable study designs is needed to more accurately evaluate the overall effect. Nonetheless, the vast majority of the posterior probability distribution indicated a positive overall effect (PP _> 0_ = 0.94).

By evaluating different aspects of study design we were able to identify study factors that appear to influence the effect size between prenatal stress exposure and offspring glucocorticoids. In particular, experimental designs elicited greater effect sizes than observational designs. Based on prior studies^7,25–28^, we would predict that organisms would adjust their physiology and behavior in response to maternal experience of chronic as opposed to more randomly dispersed, acute stressors, the latter of which might be less reliable indicators of environmental quality. It is therefore possible that experimental studies, which are purposefully designed to induce a strong impact on maternal and/or offspring physiology, tend to be severe enough that this signal is more reliably passed on to offspring. As an example, exogenous administration of glucocorticoids to eggs is unsurprisingly associated with increased glucocorticoid levels in offspring^29–31^. Observational studies of perceived stress, however, do not always measure prenatal glucocorticoid exposure directly^32,33^, and therefore inconsistent results may result from offspring in observational studies being exposed to lower glucocorticoids prenatally.

In contrast to baseline or peak reactivity, we found that examining offspring glucocorticoid recovery following stressor exposure elicited the greatest effect sizes. Several factors could be responsible for this finding. First, we identified inconsistency in the number and timing of recovery measurements reported across studies. For example, Montano *et al*.^34^ assessed corticosterone recovery to stress in mice at hours 4, 8, 12, 16 and 24, respectively, while Ping *et al*.^33^ assessed cortisol recovery of human infants at 20 and 45 minutes after a stressor, respectively. When multiple values were reported for recovery, we selected the time point with the greatest difference between cases and controls. Because of this, our methodology selected for recovery measures to have the largest possible effects. Given variation in the timing of glucocorticoid measurements across studies, it is possible that some studies simply did not detect the highest peak glucocorticoid response after a stressor with their sampling scheme, or - similarly - that some studies did not measure baseline appropriately. That said, the influence of such flaws on the magnitude of effect sizes should be minimized as each study compared a stressed group to a control group and assayed baseline and reactivity in both groups using the same approach.

Second, there may be functional reasons why glucocorticoid recovery is more strongly associated with prenatal stress exposure than other measures. Specifically, differences in cortisol recovery following stress exposure may reflect the maladaptive impacts of increased allostatic load^35^. Repeated or chronic activation of physiological systems and their attempt to maintain homeostasis can result in “wear and tear”, which may then be indexed by a prolonged response due to a delayed or inefficient return to baseline, i.e. recovery^36^.

As mammalian mothers are able to directly influence offspring biology across both gestation and lactation^37^, we predicted that maternal effects would be stronger in mammals. However, we found no significant differences between mammals and non-mammals. In addition, we might anticipate that organisms with slower life history strategies (as indicated by larger size and giving birth to fewer, higher quality offspring) would have stronger evidence for maternal effects than species characterized by faster life histories^38^. Surprisingly, none of the life history variables were unequivocally associated with effect sizes. This suggests that species with fast or slow life histories have a similar capacity for prenatal stress-induced maternal effects. These findings are therefore consistent with an ancient vertebrate origin of HPA-axis programming, and suggest that such effects may have been selected for to mediate intra-specific variability in life history strategy^13^. Given the similarities in programming capacity across vertebrates, this suggests that both mammalian and non-mammalian model organisms may be appropriate for developing a detailed understanding of prenatal programming of HPA-axis function in humans.

Studies that have directly compared glucocorticoid levels in fish^39^ and bird eggs^40^ have recorded substantial variation both within and between species. These results, like ours, suggest that there are examples of prenatal sensitivity to stress in a broad range of species, though they are only detected under certain conditions. In particular, previous studies have found that timing of breeding, laying order, offspring sex, life history stage at assessment, and types of treatment can all affect glucocorticoid programming effects^39,40^. Both the universality and specificity of this programming response further suggests a potential adaptive function of maternal stress induced impacts on offspring glucocorticoids.

Despite the strength of this analysis, principally the evaluation of the impacts of prenatal stress using state-of-the-art statistical methods that account for all non-independencies and moderating factors, there were several limitations. First, the wide range of study designs made it difficult to directly compare severity and timing of stressors across species. In order to combat this, we attempted to define our variables as broadly as we could to avoid misclassification (as described in Table 1), and we have supplied raw data in our supplementary materials to facilitate future analyses. Second, though there is good evidence that glucocorticoids induce similar phenotypes in non-mammalian species (for example, higher glucocorticoids in fish eggs are associated with smaller offspring size^39^ similar to humans and other mammals), there were relatively few studies in non-mammals included here, which diminished our ability to detect broad phylogenetic patterns. More research is needed, but the limited findings suggest that the low phylogenetic influence on effect sizes might hold up in larger comparative samples. Third, our study focused specifically on glucocorticoids. Epigenetic marks, transport proteins, and hormone receptors also respond to environmental variability and therefore could be influenced by prenatal stress and shape offspring development^41,42^. Therefore, it is possible that prenatal stress impacts HPA-axis function through these pathways instead of (or in addition to) solely the effects of prenatal stress on glucocorticoids^17,43–45^.

Species in which the maternal environment most accurately predicts offspring environment are expected to have the most consistent examples of maternal effects^10^. It is difficult to study environmental predictability across species; this could also account for the somewhat ambiguous results reported here and in another maternal-effects meta-analysis^46^. Indeed, environmental predictability may be better studied in comparisons of different populations of the same species, or studies of the same population across time, such as the hare study described above^7^.

The studies in this meta-analysis included stressors that varied widely in terms of timing, chronicity, severity, and ecological relevance. If prenatal stress-induced maternal effects are meant to be adaptive, then we would expect that stressors that are predictable or cyclical in nature would induce these effects more reliably than random, unpredictable stressors^47^. In ecological studies this is commonly assessed by studying predator density and resource availability^4^. In observational human studies, however, the stressors often included factors such as perceived racial discrimination^48^ or pregnancy-specific anxiety^49^. While potentially chronic in nature, the stressors studied in human research are nonetheless qualitatively different than those studied in ecological research, making the results somewhat difficult to compare. In order to resolve these issues it is important to have more human studies in response to a broader range of stressors, as well as in non-Western cultural contexts. Understanding how prenatal exposure to stressors associated with climate change, predation, or other extrinsic mortality risk factors, for example, could provide a more appropriate model for comparing human studies with ecological models in other species. While not assessing cortisol in pregnancy, anthropologists have studied pregnant women experiencing seasonal and chronic nutritional stress among pastoralists^50^ and hunter gatherers^51^, suggesting that such studies are potentially feasible.

In sum, we found a trend toward elevated glucocorticoids in offspring exposed to prenatal stress. These effects were strongest in experimental studies and those assessing glucocorticoid recovery following stress exposure. The lack of a strong phylogenetic signal, and the fact that life history variables were not clearly associated with the response, is consistent with the interpretation that this response is evolutionarily ancient and conserved across a broad range of species.

## Methods

We first conducted a literature review to assess all human and animal studies of prenatal stress in relation to offspring glucocorticoids. Articles were identified using reference lists and online literature searches, including Web of Science and Google Scholar. Web of Science and Google scholar were both used because of the generalized nature of their databases, which was important as we are comparing multiple species of mammals, birds, and reptiles. These databases were the only ones used because of their broad scope of topics covered and large libraries. Two of the authors (M. Wilson and A. Kim) were responsible for identification, screening and removing articles that did not fit the inclusion criteria. We used the terms *maternal stress*, *prenatal stress*, *stress reactivity*, *cortisol* and *glucocorticoid* to do a general search of both databases and screened relevant articles (N = 445). We identified articles from the databases that included some or all of those keywords in the title of the article or which described studies examined HPA axis or fetal programming for further assessment (N = 214). We also read through reference lists of relevant articles and identified other possible articles for inclusion using the same strategy outlined above (N = 7). There were no language exclusions. Articles were included in our analysis if they met the following inclusion criteria. All studies had to include a prenatal exposure to either an observed or experimentally administered stressor, and/or, in the case of birds and reptiles, synthetic administration of a glucocorticoid to the mother or egg. For simplification, both cases of prenatal stress exposure and administration of synthetic glucocorticoids are referred to as “prenatal stress.” Second, all studies had to include a measure of offspring glucocorticoid function at hatching, at viable birth age, or later, as assessed through either a baseline measurement and/or a measurement of glucocorticoid reactivity. Here we limit our measure of HPA-axis function to glucocorticoids for the sake of comparability, but acknowledge that there are several different potential HPA-axis endpoints and measurements of potential interest. In addition, we chose to exclude PTSD studies^52,53^ since PTSD is relatively unique in being associated with hypo- as opposed to hyper-cortisolism. In addition, we had to exclude all articles that did not include any statistics allowing us to estimate effect sizes and whose authors did not respond to queries for more information (N = 39). Figure 1 depicts the phylogeny of species included in the study with mean effect sizes (and range) for each species and internal branches color-coded to reflect ancestral states inferred by maximum likelihood using the *phytools* package^54^. Supplementary Figure 1 shows the total number of studies included and removed at the identification, screening, eligibility and included stages as outlined by the PRISMA statement^55^.

We used Cohen’s *d* as our measure of effect size, with the correction for small sample sizes (*d’*)^56^, reflecting the difference between stressed and non-stressed subjects. Whenever possible, we calculated *d’* and its variance from reported data on number of subjects, means, and standard deviations of experimental and control groups. For studies reporting other statistics, such as *F* tests, *t* tests, or regressions, we first calculated the correlation coefficient *r* and then converted *r* into *d* using standard formulas^56^. We built a phylogeny of all species in the sample by collating recent supertrees for mammals^57^ and birds^58^, assuming a divergence time of 321 million years ago for birds and mammals, and then grafted a branch for the garter snake onto this combined tree, assuming a divergence time of 278 million years ago for birds and reptiles^59^. Life history traits were obtained from the published literature^60^, with body size and brain size available for all species, and gestation length, weaning age, age of first reproduction, fertility, and maximum lifespan for mammals only. All life history traits were log transformed and converted into *z* scores.

We then used Bayesian phylogenetic multilevel meta-analyses^61^ to estimate the overall weighted effect size and the influence of moderator variables while accounting for phylogenetic relationships and non-independence of effect sizes stemming from the same study or the same species. This method, implemented in the *MCMCglmm* package^62^ in the R statistical environment^63^, corresponds to model 11 in ref.^61^, i.e. a random effects meta-analysis that weighs effect sizes by their standard errors, and estimates variance components for phylogeny, measurement errors, and residual variance, as well as for study level and species level random effects. We first ran an intercept only model to estimate the overall weighted effect size, and then added moderators as described in the Results. All models used non-informative priors, were run for 100,000 iterations with a burnin of 20,000^64^, and convergence was confirmed visually as well as by calculating the Gelman-Rubin diagnostic (all 1) implemented in the coda package^65^.

Bayesian models produce a probability distribution (“the posterior”) for each estimated parameter (such as the overall effect size, or the difference in effect sizes between two moderators), and there are various ways to summarize these distributions^66^. Following the convention in most meta-analyses, we here report the mean +95% credible intervals, calculated as the region of the parameter space that contains 95% of the probability density, i.e. the Highest Posterior Density Interval (HPDI). In addition, we report the proportion of the distribution that is consistent with an effect, i.e. the posterior probability (PP) that an effect is different from 0 (a one-tailed probability). As such, readers can draw their own inferences probabilistically.

In order to assess publication bias we used Egger’s regression on the residuals of a full model including all moderators^21^. As a measure of heterogeneity we report the proportion of variance explained by the study- and species-level random effects as well as by the phylogeny^67^.

For readers unfamiliar with modern meta-analyses or Bayesian statistics, we recommend Card’s book^54^ as a general introduction to meta-analyses, Nakagawa and Santos’ detailed guide for biological meta-analyses^64^, which may include phylogeny and intra-specific variation, with respective R code provided in the supplement of Hadfield and Nakagawa^59^ and several recent examples^22,45,65^, and McElreath’s book^66^ as well as several recent reviews^68,69^ as introductions to Bayesian statistics.

## Electronic supplementary material

Dataset 1
